# Unravelling the Diversity of Microorganisms in Ticks from Australian Wildlife

**DOI:** 10.3390/pathogens12020153

**Published:** 2023-01-17

**Authors:** Abdul Ghafar, Nick Davies, Mythili Tadepalli, Amanda Breidahl, Clare Death, Philip Haros, Yuting Li, Peter Dann, Alejandro Cabezas-Cruz, Sara Moutailler, Angélique Foucault-Simonin, Charles G. Gauci, John Stenos, Jasmin Hufschmid, Abdul Jabbar

**Affiliations:** 1 Department of Veterinary Biosciences, Melbourne Veterinary School, University of Melbourne, Werribee, VIC 3030, Australia; 2 Australian Rickettsial Reference Laboratory, Barwon Health, Geelong, VIC 3220, Australia; 3 Research Department, Phillip Island Nature Park, P.O. Box 97, Cowes, VIC 3922, Australia; 4 Anses, INRAE, Ecole Nationale Vétérinaire d’Alfort, UMR BIPAR, Laboratoire de Santé Animale, F-94700 Maisons-Alfort, France

**Keywords:** Australia, *Rickettsia*, ticks, tick-borne pathogens, wildlife, zoonosis

## Abstract

Ticks and tick-borne pathogens pose a significant threat to the health and welfare of humans and animals. Our knowledge about pathogens carried by ticks of Australian wildlife is limited. This study aimed to characterise ticks and tick-borne microorganisms from a range of wildlife species across six sites in Victoria, Australia. Following morphological and molecular characterisation (targeting 16S rRNA and cytochrome *c* oxidase I), tick DNA extracts (*n* = 140) were subjected to microfluidic real-time PCR-based screening for the detection of microorganisms and *Rickettsia*-specific real-time qPCRs. Five species of ixodid ticks were identified, including *Aponomma auruginans*, *Ixodes* (*I*.) *antechini*, *I. kohlsi*, *I. tasmani* and *I. trichosuri*. Phylogenetic analyses of 16S rRNA sequences of *I. tasmani* revealed two subclades, indicating a potential cryptic species. The microfluidic real-time PCR detected seven different microorganisms as a single (in 13/45 ticks) or multiple infections (27/45). The most common microorganisms detected were Apicomplexa (84.4%, 38/45) followed by *Rickettsia* sp. (55.6%, 25/45), *Theileria* sp. (22.2% 10/45), *Bartonella* sp. (17.8%, 8/45), *Coxiella*-like sp. (6.7%, 3/45), *Hepatozoon* sp. (2.2%, 1/45), and *Ehrlichia* sp. (2.2%, 1/45). Phylogenetic analyses of four *Rickettsia* loci showed that the *Rickettsia* isolates detected herein potentially belonged to a novel species of *Rickettsia*. This study demonstrated that ticks of Australian wildlife carry a diverse array of microorganisms. Given the direct and indirect human–wildlife–livestock interactions, there is a need to adopt a One Health approach for continuous surveillance of tick-associated pathogens/microorganisms to minimise the associated threats to animal and human health.

## 1. Introduction

Australia is home to more than 3000 species of wild vertebrates, and new species are being added to the list frequently [1]. Many of these vertebrate hosts are susceptible to tick infestation and its harmful effects such as skin damage, toxicosis, irritation, paralysis, allergies, and anaemia [2,3]. Ticks are obligate haematophagous ectoparasites commonly associated with the transmission of infectious agents in wild and domestic animals [2]. Additionally, 17% of human infections are vector-borne, and there is an increasing incidence of tick-borne zoonoses [4,5] that has been primarily ascribed to climate change, landscape modifications, and changes in mammalian host populations due to habitat fragmentation and degradation [6,7,8,9,10]. For example, an increase in the prevalence of tick-associated Lyme disease in North America has been attributed to the increased population of the key reservoir host (i.e., white-footed mice (*Peromyscus leucopus*)) due to forest fragmentation and associated reduced mammalian diversity [11].

While risks to human and animal health due to ticks and tick-borne pathogens (TBPs) are of longstanding concern in Australia, our knowledge of specific pathogens posing such risks is limited [12]. For example, although *Borrelia burgdorferi* s.l. has not been reliably identified in Australia, anecdotal evidence supports the association of tick bites with a Lyme-disease-like illness known as the Debilitating Symptom Complexes Attributed to Ticks (DSCATT) [12,13]. Infections with *Rickettsia* (*R*.) are also common in Australia, and clinical cases of Queensland tick typhus (caused by *Rickettsia australis*) [14,15], Flinders Island spotted fever (*R. honei*) [16], and Australian spotted fever (*R. honei* subsp. *marmionii*) were reported earlier [17].

More recent investigations have demonstrated the presence of a rich diversity of microorganisms that include potentially novel pathogens in Australian ticks. For example, Tadepalli et al. [18] reported a novel *Rickettsia* sp. belonging to the spotted fever group in ticks collected from shingleback lizards (*Tiliqua rugosa*) in southern Western Australia. Similarly, some other studies have identified novel species of *Bartonella* [19,20,21], *Babesia* [22,23,24], *Borrelia* [12,23,25,26], *Ehrlichia* [27,28], *Neoehrlichia* [12,29,30], flaviruses [12,31], and reoviruses [12,32] in Australian ticks and wildlife. However, the pathogenic potential of most of these detected microorganisms for animal and human health is currently unknown and warrants further investigations.

Traditionally, PCR-based diagnostic tools have been used for the identification of TBPs. However, these conventional tools are not ideal for large-scale surveillance programs due to limitations such as the capacity to simultaneously target only a few pathogens (usually known), requiring large volumes of target nucleic acid, and consuming a longer time for testing multiple pathogens. Additionally, conventional diagnostic tools may not target important commensals or endosymbionts within ticks that play a critical role in transmitting TBPs [33,34]. Such limitations can be overcome through the use of a novel microfluidic-based technique [35] that uses a few millilitres of the DNA template to test 48 or 96 targets on a microfluidic system (BioMark™ dynamic array system, Fluidigm) [35,36,37,38]. These chips dispense 48 (or 96) samples and 48 (or 96) PCR mixes into individual wells on a microfluidic chip, thereby performing 2304 or 9216 real-time PCRs [35].

This study aimed to characterise ticks and tick-borne microorganisms from a range of wildlife species across six sites in Victoria, Australia. We used the microfluidic PCR-based technique for screening microorganisms followed by amplifying multiple loci using conventional PCRs to further characterise pathogens/microorganisms.

## 2. Materials and Methods

### 2.1. Study Area and Tick Samples

**Figure 1:**
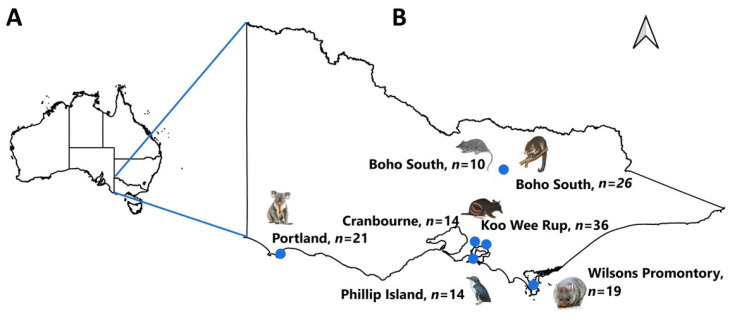
Map showing the total number of ticks collected from six different hosts from urban and regional areas of Victoria, Australia. (**A**), map of Australia; (**B**), map of Victoria.

From 2011 to 2020, tick specimens (*n* = 140) were opportunistically collected from six Australian wildlife species; i.e., agile antechinus (*Antechinus agilis*), koala (*Phascolarctos cinereus*), little penguin (*Eudyptula minor*), mountain brushtail possum (*Trichosurus cunninghami*), southern brown bandicoot (*Isoodon obesulus*), and bare-nosed wombat (*Vombatus ursinus*), across six sites (Boho South, Cranbourne, Koo Wee Rup, Phillip Island, Portland, and Wilsons Promontory) in Victoria, Australia (Figure 1; Table 1). The ticks were transferred to the Melbourne Veterinary School for identification purposes in 70% ethanol solution.

### 2.2. Morphological Identification of Ticks

Ticks were morphologically identified at the genus and species level (where possible) under a dissecting microscope (Olympus, Tokyo, Japan) following dichotomous keys [39,40]. Some of the tick specimens could not be reliably identified by using only their morphological characteristics but were subsequently identified along with other specimens via molecular characterisation as described below.

### 2.3. Molecular Identification of Ticks

A subset of representative tick specimens (*n* = 45) was selected for the extraction of genomic DNA and subsequent molecular identification in such a way that multiple specimens of each tick genus/species from each host species per location were included in the subset. Additionally, DNA was also extracted from those specimens (*n* = 20) for which morphological characterisation to the species level was not possible. Briefly, each tick was longitudinally cut into two halves; one half was preserved in ethanol and the other half was washed thrice (45 min each time) in distilled water. Subsequently, finely chopped pieces of washed specimens were subjected to genomic DNA extraction using a DNeasy Blood and Tissue Kit (Qiagen, Hilden, Germany) following the manufacturer’s protocol except that the 56 °C incubation step with proteinase K digestion lasted for 24–48 h. The quality and concentration of the extracted DNA were assessed using a spectrophotometer (ND-1000 UV–vis spectrophotometer v.3.2.1; NanoDrop Technologies, Inc., Wilmington, DE, USA) and stored at −20 °C until further testing.

All tick DNA samples were subjected to PCR amplification of the partial fragments of mitochondrial cytochrome *c* oxidase subunit I (*cox*1) and 16S ribosomal RNA (16S rRNA) loci separately using published primers [41,42] in a T100 thermal cycler (BioRad, Hercules, CA, USA). Amplification reactions were carried out in 25 µL reaction volumes containing 6.25 pmol of each primer, 3.12 mM of each deoxynucleotide triphosphate (dNTP), 5X Green GoTaq flexi reaction buffer (5 µL), 75 mM (for 16S rRNA), 25 mM (for *cox*1) of MgCl2, and 1 U of DNA polymerase (Promega, Madison, WI, USA). All reactions included known positive (DNA of *Hyalomma anatolicum* and *Rhipicephalus microplus*) and negative (UV-sterilised Milli-Q water) controls. Amplicons (5 µL) were separated on 1.5% (*w/v*) agarose gels stained with GelRed (Biotium, Fremont, CA, USA) and visualised on a GelDoc system (BioRad).

### 2.4. Microfluidic Detection of Microorganisms in Tick DNA Samples

The individual tick DNA samples (*n* = 45) were subjected to microfluidic real-time PCR for amplification of specific regions of DNA of the target microorganisms using a 48.48 dynamics array in a Bio-Mark™ real-time PCR system (Fluidigm, San Francisco, CA, USA), as described previously [35,37,38]. The details of the tested microorganisms and targeted genomic markers are given in Supplementary Table S1. Negative (no DNA), microorganism spike control (DNA of *Escherichia coli*, EDL933 strain), and tick DNA extraction controls were included in each microfluidic chip to ensure the efficient and valid amplification/detection of targets as described previously [35,37]. Microfluidic PCR results were confirmed (where only genus-level identification was achieved) using conventional PCR followed by Sanger sequencing as described previously [37].

### 2.5. qPCR Detection of Rickettsia spp.

The tick DNA samples were also screened for *Rickettsia* spp. using a real-time qPCR assay targeting citrate synthase gene (*gltA*) sequences as described by Tadepalli et al. [18]. A subset (*n* = 7) of ticks DNA-positive for *Rickettsia gltA* qPCR was further examined as described previously [18,43]. Briefly, the longer fragments of genes encoding (i) *gltA*, (ii) outer membrane protein A (*ompA*), and (iii) the 17 kDa outer membrane antigen were amplified [18].

### 2.6. DNA Sequencing and Phylogenetic Analyses

Conventional PCR amplicons generated for ticks and target microorganisms were purified using shrimp alkaline phosphate and exonuclease I (Thermo Fisher Scientific, Melbourne, Australia) and subjected to Sanger sequencing using PCR primers. Nucleotide sequences obtained for target loci of ticks (*cox*1 and 16S rRNA) and tick-borne microorganisms/pathogens (outer membrane protein B (*ompB*), *ompA*, *gltA,* and 17 kDa) were assessed for various quality parameters (such as the desired sequence length, base quality score, etc.) and assembled in Geneious Prime 2019.0.4 software using the de novo assembly function (Biomatters Ltd., Auckland, New Zealand; www.geneious.com). Unique sequences for each locus were identified via the “find duplicates” function in Geneious. Subsequently, these unique sequences were subjected to online searching within the National Center for Biotechnology Information (NCBI) (https://blast.ncbi.nlm.nih.gov/Blast.cgi) database to match their identities with known published reference sequences. All protein-coding nucleotide sequences were assessed for open reading frames, and then all unique nucleotide sequences were submitted to the NCBI GenBank. Pairwise comparisons were also conducted for each sequence dataset using BioEdit [44]. The reference sequences for each locus (of ticks or tick-borne microorganisms) were retrieved from GenBank and aligned in MEGA 11 [45] using MUSCLE v.3.8.31 [46]. Aligned sequences were trimmed to uniform lengths of 612 (*cox*1), 370 (16S rRNA), 352 (*gltA*), 434 (*ompA*), 391 (17 kDa), and 267 (*ompB*) bp.

Phylogenetic analyses were performed on aligned sequence datasets of individual loci for ticks (*cox*1 and 16S rRNA) and tick-borne microorganisms (*ompA*, *ompB*, *gltA*, and 17 kDa) using Bayesian Inference (BI), Maximum Likelihood (ML) and Neighbour Joining (NJ) methods. The NJ and ML analyses were performed in MEGA 11, whereas BI was performed using the MrBayes plugin [47] in Geneious. The jModelTest v.3.7 [48] was used to estimate the best-fit evolutionary models for individual sequence alignments based on the Akaike information criteria (AIC). The best-fit models for nucleotide sequences of ticks included the Tamura-Nei with Gamma distribution (*cox*1) and Tamura 3-parameter with Gamma distribution (16S rRNA) [49,50]. For nucleotide sequences of the microorganisms, the best-fit models were the Tamura 3-parameter (*ompB* and *gltA*), Tamura 3-parameter with Gamma distribution (*ompA*), and Kimura-2 parameter (17-kDa) datasets [49,51]. The nodal supports in the ML and NJ phylogenies were tested using the bootstrap method (10,000 replicates,) whereas the posterior probabilities of the BI analyses were calculated for 2,000,000 generations (ngen = 2,000,000) while saving every 200th tree (samplefreq = 200). Corresponding reference sequences of *Ornithodoros moubata* (GenBank accession: KJ133594), *Rickettsia bellii* (LAOIO1000001) and *R. felis* (CP000053, AF210692 and AF210694) were used as outgroups for the phylogenetic trees of ticks and microorganisms, respectively.

## 3. Results

### 3.1. Morphological and Molecular Characterisation of Ticks

The morphological examination of ticks (*n* = 140) revealed that the specimens belonged to two ixodid genera: *Aponomma* (*n* = 19) and *Ixodes* (*n* = 121) (Figure 1; Table 1; Supplementary Table S2). The genus *Ixodes* (*I.*) included four species (i.e., *I. antechini* = 7, *I. kohlsi* = 14, *I. tasmani* = 55, and *I. trichosuri* = 25), whereas only one species was identified within the genus *Aponomma* (*Ap. auruginans*: *n* = 19). However, 20 nymphal-stage specimens could morphologically be identified as either *I. trichosuri* or *I. hirsti*. Subsequently, these specimens were identified as *I. trichosuri* based on the genetic characterisation (*cox*1 and 16S rRNA loci). The molecular characterisation of the representative specimens of other tick species concurred with the morphological findings. *Ixodes tasmani* and *I. trichosuri* were found on four (koala, mountain brushtail possum, agile antechinus, and southern brown bandicoot) and two (mountain brushtail possum and southern brown bandicoot) host species, respectively; whereas *Ap. auruginans*, *I. kohlsi*, and *I. antechini* were found only on wombats, little penguins, and agile antechinus, respectively (Figure 1; Table 1; Supplementary Table S2).

### 3.2. Sequence and Phylogenetic Analyses of Nucleotide Sequences of Ticks

A total of 27 unique tick nucleotide sequences (*cox*1= 14; 16S rRNA = 13) were obtained for *I. trichosuri* (*cox*1 = 5; 16S rRNA = 4), *I. antechini* (*cox*1 = 2; 16S rRNA = 2), *I. tasmani* (*cox*1 = 3; 16S rRNA = 4), *I. kohlsi* (*cox*1 = 3; 16S rRNA = 2), and *Ap. auruginans* (*cox*1 = 1; 16S rRNA = 1). Upon an NCBI Blast search of *cox*1 sequences, the *I. trichosuri* sequences showed the highest similarity (99.5–99.7%) with the previously published sequences of the same species (MN686562, MN686563, and MN686568); that of *I. kohlsi* was identical to a sequence of *I*. *eudyptidis*/*kohlsi* (KM488522); and one sequence of *Ap. auruginans* had a similarity of 99.8% to that of the *Bothriocroton* sp. isolate (KM821511), all of which were reported from different hosts in Australia. The *cox*1 sequences of *I. tasmani* showed a 90.4–98.04% similarity to those of the same species (KX676867 and MN106731) from Australia. There was no reference sequence available for *I. antechini* in the GenBank; the sequences determined herein showed the highest similarity (91.33%) to that of *I. brunneus* (KX360364) from Canada.

For the 16S rRNA sequences, *I. kohlsi* had the highest similarity (99.7%) to those of *I. eudyptidis*/*kohlsi* (KM488490 and KM488491) from Australia, while the *I. antechini* sequences showed the highest similarity (97.1%) to a sequence (AF113929) of the same species from an unknown origin. No 16S rRNA reference sequence was available for *Ap. auruginans* and *I. trichosuri*; however, their sequences determined herein showed the highest similarities (90.4% and 92%, respectively) to those of *Bothriocroton concolor* (JN8663727) and *I. holocyclus* (MH043264), respectively, from Australia. In concordance with the BLAST search results obtained for the *I. tasmani cox*1 sequences, the 16S rRNA sequences also showed a wide range of similarities (87.75–98.27%) with those reported previously (U95906 and MH043271). The pairwise comparisons of the 16S rRNA and *cox*1 sequences of *I. tasmani* isolates characterised in this study also showed a high level of intraspecific variation (16S rRNA: 0.3–14.3% and *cox*1: 2–11.3%) (Supplementary Tables S3 and S4).

**Figure 2:**
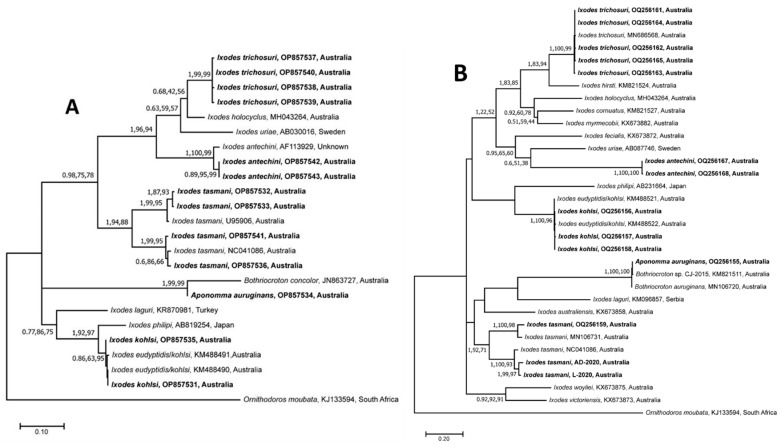
Genetic relationships of 16S rRNA gene (**A**) and cytochrome *c* oxidase subunit I gene (**B**) sequences of ticks collected from Australian wild animals from Victoria. The 16S rRNA (370 bp) and *cox*1 (612 bp) datasets were analysed using Neighbor Joining (NJ), Maximum Likelihood (ML) and Bayesian Inference (BI) methods. There was a concordance among the topology of the BI, ML, and NJ trees (not shown); only the ML tree is presented here. Nodal support is given as a posterior probability of BI and bootstrap values for NJ and ML. Sequences obtained in this study are shown in bold fonts. The trees were rooted using *Ornithodoros moubata* as an outgroup. Each scale bar indicates the number of inferred substitutions per site.

Separate phylogenetic trees for the 16S rRNA and *cox*1 sequences estimated using the three methods (BI, ML, and NJ) showed identical tree topologies; therefore, only ML trees are presented along with posterior probabilities (pp) and bootstrap support values (bs) for BI, and NJ and ML, respectively (Figure 2A,B). For both 16S rRNA and *cox*1, the clustering of sequences was similar and showed only minor differences potentially related to the unavailability of 16S rRNA and *cox*1 reference sequences for *I. trichosuri* and *I. antechini*, respectively. The *cox*1 sequences of *I. trichosuri* grouped with the previously published sequence (MN686568) of *I. trichosuri* from Australia with strong nodal support (pp = 1; bs for NJ = 100%; bs for ML = 99%) (Figure 1B), whereas the corresponding 16S rRNA sequences clustered together as a separate clade with strong nodal support (pp = 1; bs for NJ = 99%; bs for ML = 99%) (Figure 1A).

Similarly, the 16S rRNA sequences of *I. antechini* grouped with a previously published sequence (AF113829) of the same species, whereas the *cox*1 sequences formed a separate cluster with strong nodal supports (16S rRNA: pp = 1, bs for NJ = 100%, and bs for ML = 99%; and *cox*1: pp = 1, bs for NJ = 100%, and bs for ML = 100%) (Figure 1). The sequences of *I. kohlsi* grouped with previously published sequences of *I. eudyptidis*/*kohlsi* (16S rRNA: KM488490 and KM488491; and *cox*1: KM488521 and KM488522) with low to strong nodal support (16S rRNA: pp = 0.86, bs for NJ = 63%, and bs for ML = 95%; and *cox*1: pp = 1, bs for NJ = 100%, and bs for ML = 96%). Both the 16S rRNA and *cox*1 sequences of *I. tasmani* produced two distinct subclades with low to strong nodal support (16S rRNA: pp = 1, bs for NJ = 94%, and bs for ML = 84%; and *cox*1: pp = 1, bs for NJ = 92%, and bs for ML = 71%) (Figure 1). Within each subclade, the sequences determined in this study were grouped along with the corresponding reference sequences (16S rRNA: U95906 and NC041086; *cox*1: MN106731 and NC041086) of the same species previously published from Australia with strong nodal support (subclade 1—16S rRNA: pp = 1, bs for NJ = 99%, and bs for ML = 95%; and *cox*1: pp = 1, bs for NJ = 100%, and bs for ML = 98%/subclade 2—16S rRNA: pp = 1, bs for NJ = 99%, and bs for ML = 95%; and *cox*1: pp = 1, bs for NJ = 100%, and bs for ML = 93%). Both sequences of *Ap. auruginans* clustered with the respective sequences of ticks of the *Bothriocroton* genus with strong nodal support (16S rRNA: pp = 1, bs for NJ = 99%, and bs for ML = 99%; and *cox*1: pp = 1, bs for NJ = 100%, and bs for ML = 100%).

### 3.3. Microfluidic Detection of Tick-Borne Microorganisms

**Figure 3:**
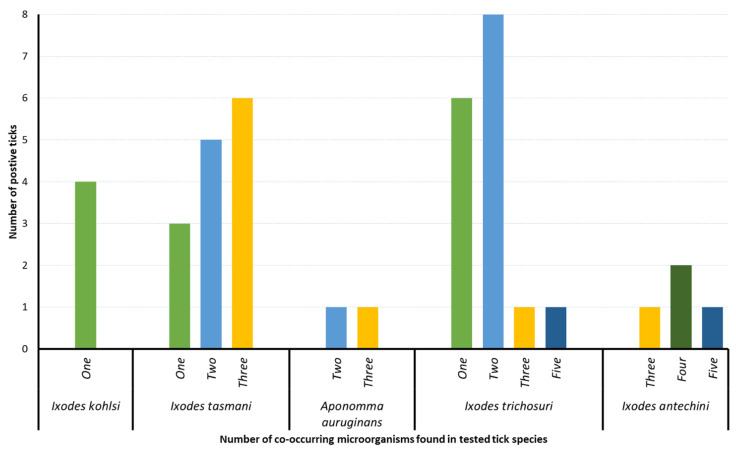
The number of co-occurring microorganisms (along *x-axis*) found in tested ticks (number provided along *y-axis*) of each species.

The microfluidic real-time PCR detected the DNA of at least one of the targeted microorganisms in 88.9% of tested ticks (40/45), and a total of seven microorganisms were detected (Figure 3; Table 2; Supplementary Table S2). The most common microorganisms detected were Apicomplexa sp. (84.4% (38/45)) followed by *Rickettsia* sp. (55.6% (25/45)), *Theileria* sp. (22.2% (10/45)), and *Bartonella* sp. (17.8% (8/45)). A low proportion of ticks was also found to be positive for the DNA of *Coxiella*-like sp. (6.7%), *Hepatozoon* sp. (2.2%), and *Ehrlichia* sp. (2.2%). (Table 2; Supplementary Table S2). *Ixodes tasmani*, *I. trichosuri,* and *I. antechini* ticks were positive for five (out of seven) detected microorganisms, whereas one and three were found in *I. kohlsi* and *Ap. auruginans* ticks, respectively (Table 2). Ticks collected from mountain brushtail possums, southern brown bandicoots, and agile antechinus had the highest number of microorganisms (*n* = 5) followed by those from bare-nosed wombats and koalas (*n* = 3) and little penguins (*n* = 1) (Table 2; Supplementary Table S2). The number of microorganisms detected in a tick species also varied in different host species. For example, *I. tasmani* ticks were positive for five, three, two, and one microorganisms in specimens collected from southern brown bandicoots, koalas, agile antechinus, and mountain brushtail possums, respectively. Similarly, *I. trichosuri* were positive for five and two microorganisms in specimens collected from mountain brushtail possums and southern brown bandicoots, respectively (Table 2).

### 3.4. Co-Occurrence of Microorganisms

The DNA of single microorganisms was detected in 13 ticks (*I. kohlsi*, *I. tasmani,* and *I. trichosuri*), 27 ticks tested positive for DNA of multiple microorganisms, and only 5 ticks were found to be negative for DNA of all microorganisms tested herein (Figure 3; Supplementary Table S2). Among ticks positive for multiple microorganisms, most of them carried two (*n* = 14), three (*n* = 9), four (*n* = 2), and five (*n* = 2) microorganisms. The highest numbers of co-occurring microorganisms were detected in *I. trichosuri* and *I. antechini* ticks, which tested positive for five microorganisms (Figure 3; Supplementary Table S2).

### 3.5. Genetic Relationship of Rickettsia Species

The BLAST search revealed variable results for different sets of the rickettsial nucleotide sequences determined herein (data not provided). The *ompA* and *ompB* sequences showed the highest similarity to those of *Candidatus R.* antechini (DQ372955; 100%) and *Candidatus R.* tasmanensis (GQ223393; 98.88%), respectively, that were previously published from Australia. Similarly, the 17 kDa sequence showed the highest similarity to that of *R. honei* (AF060704; 99.49%) from Australia, and the *gltA* sequence showed the highest similarity (99.72%) to that of *R. conorii* subsp. *raoultii* from China.

**Figure 4:**
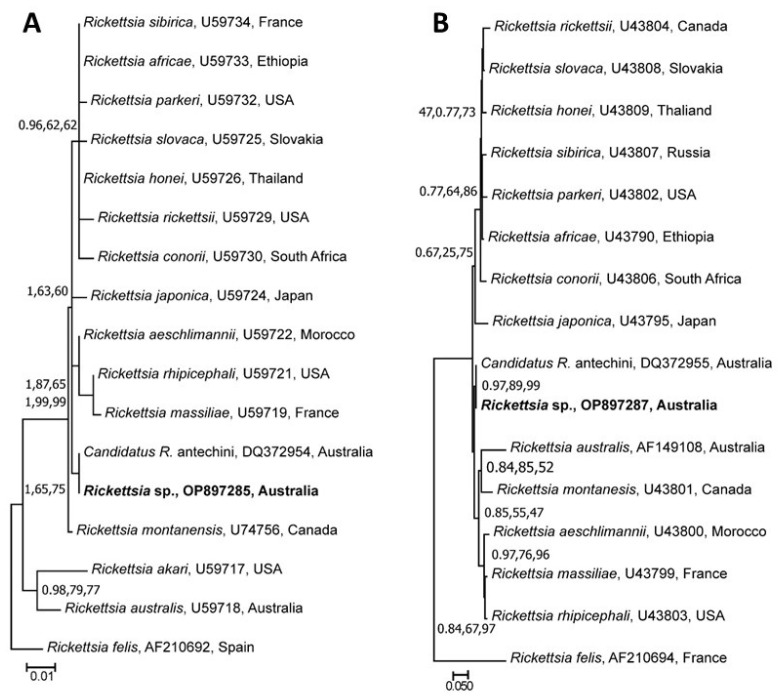

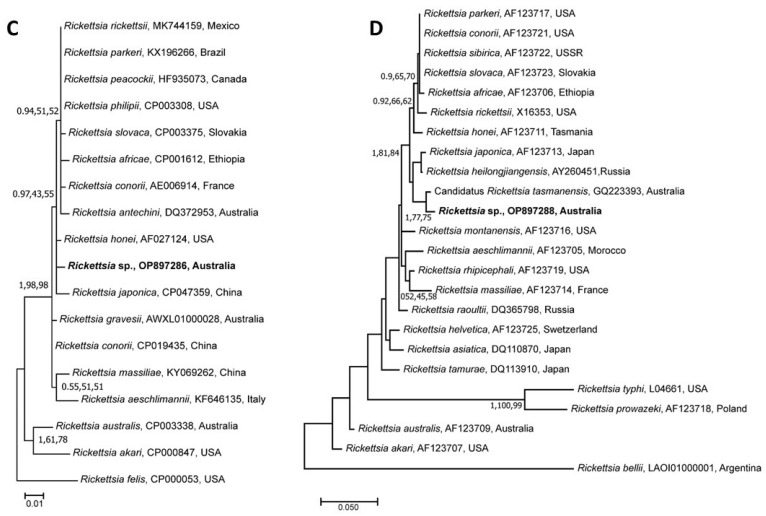
Genetic relationships of the citrate synthase (*gltA*) gene (**A**), outer membrane protein A (*ompA*) gene (**B**), 17 kDa gene (**C**), and outer membrane protein B (*ompB*) gene (**D**) sequences of *Rickettsia* sp. detected in ticks collected from Australian wild animals from Victoria. The sequence data (in bp) (352 (*gltA*), 434 (*ompA*), 391 (17 kDa), and 267 (*ompB*)) for each locus were separately analysed using Neighbor Joining (NJ), Maximum Likelihood (ML), and Bayesian Inference (BI) methods. There was a concordance among the topologies of the BI, ML, and NJ trees (not shown); only ML trees are presented here. Nodal support is given as a posterior probability of BI and bootstrap values for NJ and ML. Sequences obtained in this study are shown in bold fonts. Trees were rooted using *R. felis* and *R. bellii* as outgroups. Each scale bar indicates the number of inferred substitutions per site.

The phylogenetic analyses of unique sequences of four different genetic markers (*ompA*, *ompB*, *gltA*, and 17 kDa) of *Rickettsia* sp. determined herein also produced different topologies (Figure 4). Separate phylogenies based on *gltA* and *ompA* sequences showed that the rickettsial sequences determined in this study were grouped with respective sequences of *Candidatus R. antechini* (DQ372954, DQ372955) with various nodal supports (*gltA*: pp = 1, bs for NJ = 65%, and bs for ML = 75%; and *ompA*: pp = 0.97, bs for NJ = 89%, and bs for ML = 99%), respectively (Figure 4A,B). For the phylogenetic tree based on the 17 kDa gene, the sequences determined herein grouped separately along with *R. honei* and *R. japonica* with low nodal support (pp = 0.97, bs for NJ = 43%, and bs for ML = 55%) (Figure 4C). The genetic relationships of the *ompB* gene sequences revealed their grouping with those of *Candidatus R.* tasmanensis (GQ223393) from Australia with low to strong nodal support (pp = 1, bs for NJ = 77%, and bs for ML = 75%) (Figure 4D).

## 4. Discussion

In this study, we utilised, for the first time, a high-throughput microfluidic PCR-based technique to simultaneously detect bacterial, rickettsial, and protozoal microorganisms in ticks collected from six wild animal species in Australia. We found a diverse range of microorganisms, including several that were potentially pathogenic and of zoonotic importance (e.g., species of *Rickettsia* and *Bartonella*). The simultaneous detection of DNA of up to seven species of potentially pathogenic microorganisms highlighted the significance of such studies for the surveillance of ticks and TBPs from a One Health perspective. The molecular characterisation of *I. tasmani* revealed genetic variation among different isolates, which indicated the potential presence of cryptic species. Furthermore, the findings of our study added significantly to the growing literature on the diversity of ticks and tick-borne microorganisms of Australian wildlife.

Given that the scope of this study was limited to DNA-based detection of microorganisms, it did not exclude the possibility of the occurrence of RNA-based microorganisms (such as retroviruses), which have been reported previously in Australian ticks [12,32]. It should also be noted that no corresponding serological testing of wildlife hosts was conducted for the detection of infections. Therefore, the evidence described in this paper did not imply that there was a transmission of these microorganisms between ticks and their hosts. However, these ticks may serve as reservoirs or vectors of infection for wildlife hosts, domestic animals, and human populations.

Microfluidic-based real-time PCR is a powerful screening tool that has the capacity to perform thousands of parallel real-time PCRs simultaneously, thereby automating rotework and reducing the costs of time, labour, and consumables [35,36]. In conventional molecular amplification assays, there are a restricted number of targets that can be chosen at a time, thus limiting the screening of TBPs to targeting only pathogens (primarily those that were previously reported as being transmitted by the ticks in an area) [52]. Moreover, the very small volume of DNA template (1.3 µL) required for testing either 48 or 96 targets allows for a far greater variety of pathogens to be tested in tick DNAs (which is usually very-low-yield DNA) [35]. It is also more useful to comprehensively identify co-occurring microorganisms in ticks in situations where conventional PCR and Sanger sequencing lack capacity [22]. While next-generation sequencing is also high-throughput and exhaustive, it ultimately requires time and labour, larger volumes of templates, more optimisation steps, and complex data analyses [53,54]. The ease of application and subsequent associated analyses of microfluidic technology makes it highly suitable for use in field-based rapid diagnostics and point-of-care testing in hospital settings [55].

The high level of intraspecific sequence variation (pairwise nucleotide differences: 16S rRNA = 0.3-14.1%; *cox*1: 2-11.3%) and phylogenies based on the 16S rRNA and *cox*1 sequences of ticks demonstrated that the *I. tasmani* sequences determined herein either belong to two distinct species or that there are subspecies. These findings concurred with those of previous studies that also reported that *I. tasmani* might be a cryptic species or that there could be subspecies [40,56]. Further sampling and molecular investigations that employ longer read sequencing such as complete mitochondrial and/or whole genome sequencing would be useful to resolve the unclear phylogeny for this tick species.

It has been previously demonstrated that tick infestations lead to irritation, ill-thrift, and anaemia in wild animals such as koalas [57] and bandicoots [58]. However, little is known about the pathogenicity of the diverse array of microorganisms reported within Australian ticks in this study or in previous studies [12,29]. Certain species of *Anaplasma*, *Babesia,* and *Ehrlichia* have been known to cause a clinical disease characterised by severe anaemia, lethargy, neurologic signs, and death in kangaroos [59,60] and wild canids [61]. These pathogens along with *Theileria* sp. are also a concern in agricultural losses because they are capable of causing haematological disease and associated production losses in Australian cattle [62,63,64,65]. However, these diseases are primarily limited to bovines inhabiting northern tropical regions of Australia and are usually transmitted by cattle ticks, including *Haemaphysalis longicornis* [62] and *Rhipicephalus microplus* [65]. There is a need to test the competency of ticks characterised herein for vector potential, particularly in Victoria where other tick vectors are non-endemic.

The detection of the DNA of *Hepatozoon* sp. in 2.2% of the ticks tested in the present study underpinned the potential role of this tick in the transmission, prevalence, and consequent spillage of hepatozoonosis to domestic and wild canids. Several *Hepatozoon* species (e.g., *H. canis*, *H. banethi* n. sp., and *H. ewingi* n. sp.) have been previously reported in ticks and wildlife in Australia [22]. Hepatozoonosis, which is caused by the apicomplexan blood parasite *H. canis*, was first reported in 1905 in dogs from India and is transmitted by the ingestion of infected tick vectors [66,67]. Until now, autochthonous infections of *H. canis* have been reported in more than 60 countries across Africa, Americas, Asia, Europe, and the Middle East. Recently, an *H. canis* infection with a potential tick origin was detected for the first time in a dog in Australia [68]. Our findings and previous reports of *H. canis* in Australia could be highly significant because following initial reports of autochthonous infections of this pathogen from Germany, Austria, Slovakia, and the Czech Republic, it was later demonstrated that *H. canis* was actually endemic in these countries [22,68]. Such findings of *H. canis* in previously non-endemic countries could be attributed to the invasion of infected ticks and canids in these countries as well as the non-existence of TBP surveillance systems [68,69]. Although the DNA of this pathogen has been previously demonstrated in several tick genera including *Amblyomma*, *Dermacentor*, *Haemaphysalis* and *Ixodes*, its primary vectors are members of *Rhipicephalus sanguineus* s.l. [68]. Given the availability of tick vectors for this pathogen in Australia, further investigations are needed to determine its level of spread and associated risks.

*Coxiella burnetii*, the cause of Q fever, is usually carried by various tick species found on macropods, domestic animals, bandicoots, and birds [10]. Infections with *C. burnetii* in these animals usually remain subclinical [10], but the clinical disease has been reported to cause significant production losses in sheep [64]. This pathogen is also of high zoonotic significance because it may be transmitted to people working closely with animals, such as farmers, abattoir workers, or veterinary staff [70]. Notably, the *Coxiella*-like sp. DNA detected in 6.7% of ticks tested in the present study potentially belongs to the non-pathogenic endosymbionts [71]. This significant finding highlighted the need for further exploration of the microbiota of Australian ticks because the evolutionary origin of highly pathogenic and infectious *C. burnetii* from a maternally inherited endosymbiont is still unclear [71]. Additionally, *I. tasmani*, *Ap. Auruginans,* and *I. trichosuri* (species found positive for *Coxiella*-like sp. DNA) are not known to transmit *C. burnetii*. 

In the present study, 55.6% of the ticks tested positive for the DNA of *Rickettsia* species. These positive ticks included four species (*Ap. auruginans*, *I. tasmani*, *I. antechini,* and *I. trichosuri*) collected from five different host species. Previous studies reported various levels of occurrence of *Rickettsia* species in ticks from different hosts. For example, Izzard et al. [72] and Tadepalli et al. [18] found that 55% and 92% of *I. tasmani* and *Amblyomma albolimbatum* ticks were positive for rickettsial DNA, respectively. There are four known rickettsial species (including a novel subspecies) that cause clinical disease in mammals in Australia. These include *R. australis* (the cause of Queensland tick typhus), *R. honei* (Flinders Island spotted fever), and *R. australis* subsp. *marmionii* (Australian spotted fever) and *R. gravesii,* which are transmitted by several tick species [21]. The Blast analyses of the nucleotide sequences of multiple metabarcodes of the rickettsial isolates characterised herein did not provide conclusive evidence about their identity. Likewise, the phylogenies based on nucleotide sequences of four markers showed a close association of the rickettsial isolates determined herein with the spotted fever group’s rickettsial species (*Candidatus R*. tasmanensis and *Candidatus R.* antechini). These results suggested that the rickettsial isolates identified in this study might belong to novel species or subspecies of unknown pathogenic and/or zoonotic potential. However, these findings should be further investigated through comparative molecular typing, isolation, and whole-genome characterisation.

## Figures and Tables

**Table 1:** Host and location details of tick species used in this study.

Host Species	Tick Species	Location (Latitude, Longitude)	Ticks	
			Collected	Tested
Bare-nosed wombat (*Vombatus ursinus*)	*Aponomma auruginans*	Wilsons Promontory (−39.0080, 146.3895)	19	2
Little penguin (*Eudyptula minor*)	*Ixodes kohlsi*	Phillip Island (Nature Parks) (−38.4833314, 145.2333324)	14	6
Koala (*Phascolarctos cinereus*)	*Ixodes tasmani*	Portland (−38.3333, 141.6000)	21	4
Mountain brushtail possum (*Trichosurus cunninghami*)	*Ixodes trichosuri*	Boho South (−36.783, 145.800)	24	10
	*Ixodes tasmani*		2	2
Agile antechinus (*Antechinus agilis*)	*Ixodes antechini*		7	4
	*Ixodes tasmani*		3	1
Southern brown bandicoot (*Isoodon obesulus*)	*Ixodes tasmani*	Koo Wee Rup (−38.198798, 145.489126)	30	8
	*Ixodes trichosuri*		6	1
	*Ixodes trichosuri*	Cranbourne (Botanic Gardens) (−38.1298617, 45.2701999)	14	7

**Table 2:** Diversity of microorganisms found in ticks of wildlife species collected from six localities in Victoria, Australia.

Microorganisms	Little Penguin (*Eudyptula* *minor*)	Koala (*Phascolarctos cinereus*)	Bare-Nosed Wombat (*Vombatus ursinus*)	Mountain Brushtail Possum (*Trichosurus cunninghami*)		Agile Antechinus (*Antechinus agilis*)		Southern Brown Bandicoot (*Isoodon obesulus obesulus*)	
	*Ixodes kohlsi* (*n* = 6)	*Ixodes tasmani* (*n* = 4)	*Aponomma auruginans* (*n* = 2)	*Ixodes* *trichosuri* (*n* = 10)	*Ixodes* *tasmani* (*n* = 2)	*Ixodes* *antechini* (*n* = 4)	*Ixodes* *tasmani* (*n* = 1)	*Ixodes* *tasmani* (*n* = 8)	*Ixodes* *trichosuri* (*n* = 8)
Apicomplexa sp.	4	3	2	7	2	4	1	8	7
*Bartonella* sp.	-	-	-	3	-	3	-	2	-
*Coxiella*-like sp.	-	-	2	1	-	-	-	-	-
*Ehrlichia* sp.	-	-	-	-	-	1	-	-	-
*Hepatozoon* sp.	-	-	-	-	-	-	-	1	-
*Rickettsia* sp.	-	1	1	6	-	4	-	8	4
*Theileria* sp.	-	1	-	2	-	4	1	2	-

## Data Availability

The nucleotide sequences determined in this study can be accessed via NCBI GenBank accession numbers OP857531-OP857543 and OQ256155-OQ256168 (for 16S and *cox*1 sequences of ticks, respectively) and OP897285-OP897288 (*glt*A, *omp*A, *omp*B, and 17kDa sequences of *Rickettsia*).

## Supplementary Materials

The following supporting information can be downloaded at: https://www.mdpi.com/article/10.3390/pathogens12020153/s1, Table S1: Details of target genetic markers used for testing microorganisms in tick DNA; Table S2: Host and location details of ticks tested and the real-time PCR cycle threshold (ct) values for microorganisms detected in this study; Table S3: Pairwise percentage nucleotide similarities of 16S ribosomal RNA sequences of *Ixodes tasmani* determined in this study; Table S4: Pairwise percentage nucleotide similarities of *cox*1 sequences of *Ixodes tasmani* determined in this study.
