# Supplementary material for: Unravelling the Diversity of Microorganisms in Ticks from Australian Wildlife
Source: Pathogens. 2023 Jan 17;12(2):153. doi: 10.3390/pathogens12020153 (PMC9967841; doi:10.3390/pathogens12020153)
Supplement: Supplementary file 1 [file pathogens-12-00153-s001.zip › pathogens-2012514-supplementary.pdf]

Supplementary material

Table S1

Details of target genetic markers used for testing microorganisms in ticks DNA

**Target microorganism (Genus/Phylum)**	**Target microorganism (species)**	**Genetic marker (s)**
Anaplasma	*Anaplasma* (*A.*) spp.	16S
	A. marginale	*msp*1
	A. platys	*groEL*
	A. phagocytophilum	*msp*2
	A. ovis	*msp*4
	A. centrale, A. bovis	*groEL*
Apicomplexa	Apicomplexa spp.	18S
Aegyptianella	*Aegyptianella pullorum*	*groEL*
Babesia	*Babesia* (*B.*) *microti, B. bovis*	*CCTeta*
	B. ovis, B. bigemina, Babesia sp. EU1, *B. venatorum*	18S
	B. divergens, B. canis	*hsp*70
	B. caballi, B. gibsoni	*rap*1
	B. duncani	ITS2
Bartonella	*Bartonella* (*Ba.*) spp.	*ssrA*
	Ba. henselae	*pap*31
	Ba. barcilliformis	*rpoB*
	Ba. vinsonii subsp. berkhoffii	ITS
Borrelia	*Borrelia* (*Bo.*) spp.	23S
	Bo. miyamotoi, Bo. lonestari, Bo. theileri	*glp*Q
	Bo. anserina, Bo. afzelii, Bo. spielmanii	*fla*
	Bo. burgdorferi s.s., *Bo. garinii, Bo. lusitaniae, Bo. bissettii*	rpoB
	Bo. parkeri	*gyrB*
	Bo. turicatae	ITS
	Bo. valaisiana	*ospA*
Mycoplasma	*Mycoplasma* (*M*) spp., *Candidatus Mycoplasma* (*C.M.*) *haemominutumae, C.M. haematoparvumae*	16S
	M. wenyonii, M. haemocanis	23S
	M. haemofelis	*dnaK*
	M. ovis, M. suis, C.M. turicensisae	*rnpB*
Coxiella	*Coxiella* (*C.*) *burnetii*	*IS*1111, *icd*
Cytauxzoon	*Cytauxzoon felis*	ITS2

Ehrlichia	*Ehrlichia* (*E.*) spp.	16S
	E. ruminatium, Panola Mountain Ehrlichia, E. canis	*gltA*
	E. chaffeensis, E. ewingii	*dsb*
Hepatozoon	*Hepatozoon* (*H.*) spp, *H. americanum, H. canis*	18S
Francisella	*Francisella* spp.	*tul*4, *fopA*
Leishmania	*Leishmania* (*L.*) spp.	*hsp*70
	L. infantum	ITS
Rangelia	*Rangelia vitalli*	18S
Rickettsia	*Rickettsia* (*R.*) spp., *R. prowazekii*	*gltA*
	R. parkeri	*ompA*
	R. felis	*orfB*
	R. akari, R. typhi, R. andeanae	*ompB*
	R. rickettssii, R. conorii, R. slovaca, R. massiliae, R. helvetica, R. aeschlimannii	ITS
	R. africae	*sca*1
	Neorickettsia sp.	*groEL*
Theileria	*Theileria* (*Th.*) spp., *Th. velifera, Th. parva*	18S
	Th. mutans	ITS
	Th. equi	*ema*1
Wolbachia	*Wolbachia* spp.	*wsp*

Table S2

Host and location details of ticks tested and the real-time PCR cycle threshold (ct) values for microorganisms detected in this study.

Ticks	Host	Location	Lab ID	*Ehrlichia* sp.	*Rickettsia* sp.	*Bartonella* sp.	*Coxiella*-like sp.	Apicomplexa	*Theileria* sp.	*Hepatozoon* sp.
Aponomma auruginans	Bare-nosed wombat	Wilsons Promontory	11	-	-	-	+/12.38	+/19.42	-	-
			12	-	+/26.42	-	+/22.12	+/21.54	-	-
Ixodes antechini	Agile antechinus	Boho South	23	-	+/9.32	-	-	+/17.44	+/24.05	-
			24	-	+/9.53	+/19.69	-	+/5.28	+/7.66	-
			27	+/18.72	+/22.94	+/17.4	-	+/5.03	+/17.6	-
			28	-	+/24.48	+/21.53	-	+/15.2	+/19.75	-
Ixodes kohlsi	Little penguin	Phillip Island	1	-	-	-	-	+/25.4	-	-
			2	-	-	-	-	-	-	-
			3	-	-	-	-	+/24.1	-	-
			4	-	-	-	-	+/23.78	-	-
			5	-	-	-	-	-	-	-
			6	-	-	-	-	+/22.77	-	-
Ixodes tasmani	Koala	Portland	22	-	+/10.81	-	-	+/11.04	+/15.02	-
			7	-	+/23.97	-	-	+/23.67	-	-
			8	-	-	-	-	+/11.6	+/15.58	-
			9	-	-	-	-	+/22.93	-	-
Ixodes tasmani	Mountain brushtail possum	Boho South	10	-	-	-	-	-	-	-
			14	-	-	-	-	+/16.01	-	-
	Agile antechinus		25	-	-	-	-	+/19.68	-	-
	Southern brown bandicoot	Koo Wee Rup	29	-	+/8.5	-	-	+/16.18	+/19.84	-
			30	-	+/12.07	-	-	+/23.02	-	+/24.24
			31	-	+/10.66	-	-	+/17.59	+/23.07	-
			32	-	+/9.47	+/24	-	+/15.95	-	-

			34	-	+/11.08	-	-	+/27.45	-	-
			36	-	+/10.5	-	-	+/24.01	-	-
			37	-	+/9.43	+/17.54	-	+/17.15	-	-
			38	-	+/9.51	-	-	+/24.66	-	-
Ixodes trichosuri	Mountain brushtail possum	Boho South	13	-	+/24.16	-	-	+/4.42	-	-
			15	-	+/8.5	+/22.68	+/25.86	+/17.09	+/22.97	-
			16	-	+/11.62	-	-	+/23.28	-	-
			17	-	-	-	-	-	-	-
			18	-	-	-	-	-	-	-
			19	-	-	-	-	+/24.49	+/23.66	-
			20	-	+/11	-	-	-	-	-
			21	-	+/24.2	+/26.15	-	+/34.97	-	-
			26	-	-	+/27.13	-	+/22.35	-	-
			33	-	+/26.07	-	-	+/22.6	-	-
	Southern brown bandicoot	Koo Wee Rup	35	-	+/24.85	-	-	+/22.84	-	-
		Cranbourne	39	-	-	-	-	+/15.29	-	-
			40	-	-	-	-	+/22.29	-	-
			41	-	+/25.74	-	-	+/24.84	-	-
			42	-	-	-	-	+/22.84	-	-
			43	-	+/24.8	-	-	+/20.77	-	-
			44	-	-	-	-	+/33.06	-	-
			45	-	+/27.59	-	-	-	-	-

Table S3

Pairwise percentage nucleotide similarities of 16S ribosomal RNA sequences of *Ixodes tasmani* determined in this study

Sequence ID	7-2020	10-2020	AD-2020	L-2020
7-2020		99.7	85.9	86.5
10-2020			85.7	86.2
AD-2020				98.2
L-2020				

Table S4

Pairwise percentage nucleotide similarities of *cox*1 sequences of *Ixodes tasmani* determined in this study

Sequence ID	7-2020	AD-2020	L-2020
7-2020		88.7	89.2
AD-2020			98
L-2020			
